# Functional significance and welfare implications of chewing in dogs (*Canis familiaris*)

**DOI:** 10.3389/fvets.2025.1499933

**Published:** 2025-03-26

**Authors:** Rimini Quinn, Sophie Masters, Melissa Starling, Peter John White, Kathryn Mills, David Raubenheimer, Paul McGreevy

**Affiliations:** ^1^Sydney School of Veterinary Science (SSVS), Faculty of Science, University of Sydney, Camperdown, NSW, Australia; ^2^Charles Perkins Centre, University of Sydney, Camperdown, NSW, Australia

**Keywords:** telos, food acquisition, dental health, stress, chew rate, gnaw, bite force, diet

## Abstract

Dogs chew on both nutritive and non-nutritive items as part of their food acquisition, ingestive behaviour, self-care, and social interactions. Various definitions distinguish chewing from related oral activities, such as gnawing, masticating, and biting. Surprisingly, despite chewing being a ubiquitous behaviour in dogs, its relevance to a dog’s comfort, health, and purpose remains unclear. Additionally, the risk of dental fractures or other injuries may lead veterinarians to advise against feeding bones to dogs. This article explores the literature on chewing in dogs through the ethological framework of “Tinbergen’s Four Questions” and the Five Domains framework for animal welfare assessment. Evidence is gathered from wild and domestic canids and from human and animal models where shared physiological or biological processes provide insight. Chewing appears to promote biological fitness, providing benefits such as dental and oral hygiene, digestive health, bone strength, psychological health, and stress management. Furthermore, this article discusses the evolutionary importance of chewing, the mechanisms underlying bite force, chew rate and morphology, and the development of chewing throughout a dog’s life, from primary teeth eruption to senescence. Application of the Five Domains framework for animal welfare helps assess the impact of chewing, or lack thereof, on a dog’s welfare. A dog’s preference for chew items is primarily driven by odour, taste, and mouthfeel. Macronutrient proportions may also play a role in food preferences, which, in turn, can affect the selection of chewable items. A lack of preferred chew items may result in redirected chewing toward less appropriate items, such as non-food chews that could be harmful to dentition or the gastrointestinal tract (GIT). Chewing on such inappropriate items may also lead to the adoption of alternative oral behaviours or reduced their contentment by impeding telos. Overall, chewing positively impacts a dog’s physical and psychological health, contributing to its welfare and appearing essential as a regular part of a dog’s daily life. However, the significant benefits of chewing must be carefully weighed against potential risks.

## 1 Introduction

When given the opportunity, most domestic dogs (*Canis familiaris*) chew. However, there are knowledge gaps regarding the importance of this activity for companion and kennelled dogs, how preferred substrates may differ with the dogs’ size and head shape, and what constitutes a minimal daily requirement for good health and welfare. Dogs that do not achieve optimal chewing rates may be prevented from accessing their preferred substrate at their preferred access times or face a limiting factor such as dental pain. In any case, the amount of chewing needed for optimal health and the minimal chewing required for optimal welfare remain unknown. Domestic dogs (hereafter referred to simply as dogs) use their teeth for food acquisition (1, 2), grooming (3), and social interactions, including play (4) and agonistic interactions (5). Beyond the ultimate outcomes of these behaviours—such as a full stomach, a clean coat, and improved social bonds—chewing may also reflect proximate motivation, as the activity is valuable and enjoyable. Opportunities to engage in behaviours that are part of a dog’s normal comfort, ingestive, and social behavioural repertoire reflect canine telos and promote positive welfare when assessed through the Five Domains of welfare (6).

The care and husbandry of companion dogs are largely determined by the priorities of the humans who keep them rather than by the dogs themselves (7), and communication between humans and dogs may not always be cyno-centric (8). Therefore, it is important to address the resulting shortfalls by reviewing the peer-reviewed and grey literature on free-ranging, wild, and companion canids and the broader context of chew research across mammals. Evidence across species aims to bridge gaps in the canid literature. Although not without limitations, using research from other species is valuable due to similarities in physiological processes across animals, just as animal models play a role in research that is ultimately relevant to humans (9). The goal for the current review is to examine how the opportunity to chew, or the lack thereof, affects dogs’ health and welfare. This may help the guardians of companion and working dogs meet the oral needs of the canids in their care.

## 2 Definitions

It is important to define the common behavioural verbs related to oral activity and distinguish between them. The key verbs—lick, bite, gnaw, chew, and masticate—are observed in canids. Among these verbs, “gnaw,” “chew,” and “masticate” are often used interchangeably. For this review, their similarities and differences are noted (Table 1) to aid in the development of ethograms that inform what is measured in observational and mechanical studies.

**Table 1:** Definitions of terms relevant to chewing in dogs.

Oral activity verbs relevant to chewing in dogs			
Term	Definition	Example	Comments
Lick	To pass the tongue over (something) to taste, moisten, or clean it (Oxford Dictionary)	Dogs lick their coat as part of grooming and each other for grooming and social affiliation. Dams lick their puppies to stimulate defecation and urination and to stimulate arousal and bonding. Dogs lick food items to taste them and as a component of eating.	Licking is a jaw action that has an autonomic cycle, as described in rabbit models, as part of grooming. This is a different cadence to the sucking rate and chewing rate, each under autonomic control (202).
Bite	To *seize with teeth so as to enter, grip, or wound* (Merriam-Webster Dictionary). Predominantly with the canine teeth.	Dogs capture prey such as a rabbit with a bite and bite a conspecific that fails to heed preceding body language signs. A fearful dog tethered in a utility vehicle may bite a hand coming toward it.	To bite is singular and one component in a chewing or mastication sequence. It can serve a prehensile function as part of an ingestive behaviour or act as a defensive mechanism within a protective behaviour sequence.
Gnaw	To use the incisors, canines, premolars (PM), and/or molars (M) to wear away or take small bites out of an item (Oxford Dictionary)	Dogs gnaw on pruritic skin with incisors, leaving alopecia, dermatitis, and other signs of irritation. Dogs may gnaw on table legs or a stick with PMs, and Ms. Gnaw notches are used for diagnostic purposes in archaeological and forensic fields to identify the species of the gnawer and to age the gnawed cadaver (203, 204).	For this study, gnawing is the use of all teeth to produce notches (204), marks, or other signs of dismembering to a substrate and precludes prehension, formation of pulp/bolus, substrate in the oral cavity, and swallowing. Thus, the item is usually not food and is relatively resistant to wear, or the dog is using inhibition of full bite force, such as when gnawing on themselves.
Chew	To cut and crush an edible substrate with the Ms., PMs and canine teeth and rip and pull with the incisors to produce smaller or softer pieces as a precursor to swallowing for nutritive items (Cambridge Dictionary) or spat out for non-nutritive items.	Dogs chew a bone such as a bovine tibia or chew smaller prey such as a chicken carcass or turkey wing, dried fish skin or pig’s ear. They may chew with more effort on a cow hoof or antler. Dogs chew plant matter, such as a carrot, or chew to open a shell and remove the valued middle, such as a hazelnut or coconut. Dogs chew soft toys and rope toys, leaving the fluff and rope material strewn into pieces.	Non-nutritive substrates such as toys are broadly described as being chewed up, although they are not swallowed. These are dismembered into pieces by the act of chewing and then spat out (unless a problem behaviour is present, such as pica - the ingestion of non-nutritive items).
Masticate	Mix a mouthful of ingesta with saliva using the tongue and teeth to produce a bolus in preparation for swallowing (104).	Dogs masticate on a piece of meat or one mouthful of dry food, which will be swallowed.	Biologically describes the stage of digestion in the oral cavity and is always related to food (Merriam & Webster Dictionary)

This review uses the term “*chew”* to mean persistently grasping, stripping, and tearing with the canines and incisors, as well as cutting, cracking, and crushing with the molars to deconstruct a solid item into portions. The forelimbs and claws may be used to orient and hold the item, and this article will collectively refer to the paws, jaws, tongue, lips, and teeth as the “*chew apparatus*.” It will refer to all nutritive items as “*chews”* unless there is a reason to specify a particular type (e.g., bones). Chews will indicate nutritive chew items that can be swallowed, move through the digestive tract, and provide nourishment. Examples include rawhide, bones, and Dentasticks® (Mars Inc., Virginia, USA). A non-nutrient chew item is neither nourishing nor meant to be swallowed. Examples include sticks, balls, and Nylabones®, which can provide a chewing opportunity, particularly in the absence of the aforementioned chews.

For this review, the functional significance and welfare implications of chewing in dogs will be examined through two lenses: Tinbergen’s four questions and the Five Domains framework. Tinbergen’s questions facilitate a comprehensive exploration of animal behaviour from four biological perspectives (10). Simultaneously, the Five Domains framework can be used to investigate how this behaviour influences welfare. Together, these approaches allow for a comprehensive review of the functions of chewing in dogs.

## 3 Tinbergen’s questions as they apply to chewing in dogs

Tinbergen’s four questions seek to explain animal behaviour by analysing its phylogeny, adaptation, causation, and ontogeny. The phylogeny of examines describes how it originated and evolved, while function (or adaptation) explains why it occurs from a species-survival perspective, i.e., what evolutionary benefit it confers. Together, phylogeny and adaptation explain the ultimate development of chewing over generations. Causation (or mechanism) explores the biological mechanisms underlying a behaviour, while ontogeny examines its development throughout an individual’s lifetime. Together, these perspectives focus on the individual within a generation and relate to proximate motivation (11).

### 3.1 Phylogeny

The phylogeny of canine chewing examines how chewing has evolved and how the telos of contemporary dogs is reflected in their chewing behaviour. This is a fundamental scholarly step because the majority of dog populations have evolved in an anthropogenic environmental niche, meaning they developed in closer association with humans than other Canidae, such as jackals, coyotes, and wolves, would typically venture (12). Thus, dogs have evolved to rely on a human-linked environment. This niche has shaped the dogs’ evolution over at least 12,000 years, distinguishing them from other Canidae. Even free-ranging dogs, defined as those responsible for their own reproduction or food acquisition, comprise 85% of the current global dog population and (12) forage on materials offered by humans, discarded food, scavenged human food, or faecal waste (13–16). There may be exceptions; a notable one is the Australian dingo, which, at least after being established in Australia, has not undergone the same evolutionary processes as domestic dogs (17, 18).

As opportunistic omnivores that eat both animal and plant-based foods, dogs show great plasticity in their ingestive behaviour and consume a wide variety of foods, depending on availability, which can vary significantly across seasons and years in free-ranging animals (19). Dogs will consume meat-based food if available (20), suggesting that the adage “if it smells like meat—eat it” holds true (21). However, they can survive on little or no meat due to their ability to synthesise taurine (19). In addition to consuming human-sourced foods, dogs use group predation for large prey (though rarely), solitary predation for medium-to-small prey, and general foraging (22, 23). Dogs are specialists of small-prey predation, targeting a size range from small mammals, such as rabbits, to insects. In addition to predation, they forage for items such as carrion, eggs, and fruits (16, 22, 24). In contrast to hunting large prey, these methods involve a reduced energy expenditure and present a lesser risk of injury or loss from theft by larger predators (25).

Canids possess a large gape and use their canine and carnassial teeth [upper premolar (PM4) and lower rostral molar (M1)] for effective biting (26) and ripping techniques during a hunt. Premolars do not make contact, creating a carrying space along with slicing ability, as one would expect from species that use prey as a food source (27). The molars (upper M1-3 and lower distal M1 and 2) of canids are primarily used for crushing both plant and animal foods, unlike obligate carnivores such as felids, which possess no grinding molars (PM3/2 3/2 and M 1/1) (19).

Wild, free-ranging canids spend more time engaging in feeding behaviours than their companion and kennelled counterparts. For example, Australian dingoes average 26.1 min per feeding bout when consuming sambar deer carcasses (28). Furthermore, when feeding on kangaroo carcasses, dingoes spend between 52 and 80 min per feeding bout, depending on the environment (29). In contrast, captive African wild dogs (*Lycaon pictus*) spend 58.7 min feeding on whole carcasses (both kangaroo and deer), compared to only 3.2 min when given meat pieces (30). Consequently, anthropogenic diets fed to companion dogs are structured in ways that are likely to shorten their feeding period. Increasing opportunities to chew for these dogs can extend feeding periods to mimic those found in the wild.

The anthropogenic environment has exposed dogs to cooked meat, which contains volatile organic compounds that may be more attractive in scent and taste profile for dogs than raw meat (31). Cooking renders animal (and plant) food substrates more digestible than their uncooked equivalents. *Canis familiaris* has a similar gastrointestinal tract (GIT) to other Canidae, such as the red fox (*Vulpes vulpes*), African wild dog (*Lycaon pictus*), and dhole (*Cuon alpinus*), all of which share a proportionally short large intestine and long small intestine, which attenuate gut transit times (19, 32). In occupying the human niche, the dog’s GIT may have become partially modified to accommodate a cooked and mixed diet. Food transit times are also decreased by the hair and other fibre content (19), suggesting that, when chewed, the tough and fibrous animal and plant components assist in healthy gut motility. Dogs produce pancreatic *α*-amylase, which allows them to metabolise carbohydrate-laden food, as in humans and several other human-associated species (33). Complex carbohydrates, including starch, typify current anthropogenic diets (34) as well as many commercial dog chews, such as Greenies® (Mars Inc., Kansas City, USA; approximately 58% as carbohydrate)^1^ and Pedigree® Dentastix (approximately 88% as carbohydrate).^2^

In summary, dogs have the necessary apparatus for chewing, including large, strong carnassial and canine teeth and modifications to allow them to thrive on anthropogenic and omnivorous diets. Wild and free-ranging canids spend more time in food acquisition and feeding behaviours (29) than companion and kennelled dogs when fed contemporary commercial foods, which may indicate that the latter’s motivation to chew is not fully satisfied.

### 3.2 Function

Biological fitness encompass all of a species’ adaptations that contribute to its capacity to pass on its genes through reproduction and, in some cases, by assisting related individuals in reproducing. The primary functional role of chewing in the evolution of dogs is as a form of pre-deglutition food processing. An intriguing question is whether chewing has also been developed to fulfil additional functions that are central to the wellbeing of dogs during domestication or even before. This section forms a substantial component of this review, partly because of functional consideration addressing an important yet underrepresented topic in veterinary and companion animal literature (35).

#### 3.2.1 Food acquisition

Chewing is an essential part of a dog’s food acquisition and survival repertoire, following location, stalking, and prehension as a component of predatory behaviour. The targeted tissues in a carcass include muscle, integument, and internal organs, notably the liver and intestines (36), where chewing is an early step of digestion and facilitates accessing nourishment that is sequestered, including dismembering flesh from bone and shattering bones to access the marrow. Chewing also enables the swallowing of the most durable and challenging sources of nutrients, such as hard or fibrous plants (such as grasses and nuts) and animals (such as horns, hooves, and hair), which may fulfil requirements for fibre and probiotics (37, 38).

Scent determines whether food is tasted, with dogs, when given a choice, most commonly eating high-protein and high-fat diets if they approach them, but not a high-carbohydrate diet (*n* = 15) (39). Given the opportunity, dogs eat macronutrients in set proportions. A study of individuals representing five breeds found that dogs (*n* = 51) tended to select diets with a consistent protein:carbohydrate: fat (PCF) ratio of approximately 30:7:63 by energy (40). Roberts et al. (39) showed dogs would initially select a high-fat diet over high protein, but over days of food availability, fat selection reduces to form a balance with protein as an energy source, where protein is consistently selected between 25 and 35% of energy (40). These proportions are important in this discussion as we propose that they may inform preferences for chew types and affect behaviour (41) toward chews.

In addition to what dogs eat, the form of the food may be important to allow sufficient chewing as a key constituent of digestion. Many commercially available dog foods are partially digested by being processed (for example, minced meat and crushed bones)^3^ or homogenised and extruded (for example, dry food), thus reducing the requirement for chewing and removing any ability to self-select components.

#### 3.2.2 Health

Chewing, even without the value of acquiring nourishment, appears to be intimately connected to many aspects of physical health and fitness, including dental and oral health, digestion, microbiome health, cognitive function, stress management and prevention, and bone strength.

##### 3.2.2.1 Dental and oral health

The shearing forces dogs apply when they chew not only disrupt the integrity of the targeted substrate but also enhance oral and dental hygiene, ultimately limiting the development of periodontal disease (42). Periodontitis involves inflammation and infection of the tissues surrounding the teeth, with its sequelae including oral ulcers, increased pathogenic bacterial load, bleeding of the gums, loss of alveolar bone and teeth, and halitosis (43).

Dental disease in wild canids occurs in comparable sites as in dogs (44), which are local regions that are unreachable by the tongue (45) and, therefore, require abrasion by other methods, such as chewing. Prime dental disease locations are the fourth maxillary premolar (PM4) (due to its proximity to the salivary duct), the buccal surfaces of the canines and distal maxillary PMs, and the first mandibular molars (M). Although they appear less prevalent than in dogs at equivalent ages, periodontitis, calculus, caries, alveolar bone loss, and dental fractures have been reported in wild canids. Dubravka et al. (44), who studied dentition in the skulls of 34 wolves (*Canis lupus*), found only three (8.8%) of that sample had dental disease, of which two had a fractured tooth that would have, in itself, affected dental hygiene.

Dental disease appears to be more prevalent in captive than free-ranging wild canid species. The prevalence and severity of dental disease were greater in captive maned wolves (*Chrysocyon brachyurus, n* = *38*) fed various human-produced diets than in free-ranging maned wolves (*n* = 80) (46) that subsisted on small prey. Similarly, dental disease was reported in a small study of captive wolves (*Canis lupus*, *n* = 4) in which two were fed a soft (meat) diet, and two were fed an extruded dry food diet. Both diets were deemed nutritionally balanced and included bone meal. The wolves were given these diets for 4 months after a polishing procedure, so they started the study with comparable teeth. Both groups accumulated plaque, most commonly on the maxillary PM4, where 34.7% of the tooth area was plaque-covered in the meat-fed wolves, and 27% was plaque-covered in the dry-food-fed pair (45). This suggests dietary form and the requirement to chew, not only nutrient content, play a role in oral and dental health in canids.

A Characteristic of dogs with dental disease may by their reliance on humans for their food. When the need to chew for food acquisition is overlooked, as observed in dogs fed processed diets, they crumble easily in the mouth (47), and the risk of dental disease increases (48). The prevalence of periodontal disease (using visual dental scores) has been reported at 86.3% among companion dogs in commercial breeding facilities in the US (49) and 89% of dogs that attended a veterinary clinic in Albania (50). In the UK, 12.5% of dogs that attended veterinary clinics over 12 months required immediate dental treatment (51). Rosenburg et al. (205) found that, by 26 months of age, 95% of a laboratory beagle colony (*n* = 125) fed a pelleted dog food had dental disease.

From approximately 1 year of age, dental disease is positively associated with age and inversely associated with body weight (49, 51). The reported beneficial effects of chewing relate to less periodontal disease, halitosis, plaque, and calculus accumulation. Harvey et al. (42) found that chewing had a protective effect on dental health, which increased as the range of chewing options increased, while decreased masticatory activity is a risk factor for splaque accumulation (52). In a 14-day study examining dogs offered two types of bone, Marx et al. (53) found that chewing epiphyseal bone (or spongy bone, SB, found in the rounded ends of long bones) initially removed calculus from the labial surface of PM and M teeth more effectively than chewing harder cortical bone (CB) (*n* = 8). This effect may be due to greater tooth surface penetration into the softer SB compared to the harder CB, particularly in the early stages of chewing before the bones begin to splinter.

Another study of chew-deprived adult dogs (4 years old, *n* = 12) found that chewing 4 cm autoclaved bovine femur fragments for 13 days (20-h access per day) reduced dental calculus and gingival inflammation compared to their pre-test levels. After 3 days of chewing, the SB group showed a 57.7% reduction in calculus coverage, whereas the CB group, only demonstrated a 35.2% reduction. However, over the entire of the study, both bone types resulted in almost a 90% reduction in calculus coverage. Some dental diets and chews are formulated to be nutritionally balanced, but many are marketed as supplementary to a balanced diet and may not meet pet food standards.^4^ Studies have shown that dental chews can reduce the progression of plaque, calculus, and halitosis when administered daily as a supplement to dry food, compared to control dogs (*n* = 60) fed dry food only. Both test and control groups developed plaque over the 28-day testing period, with test dogs accumulating 32% less plaque than control dogs (54).

These findings highlight that bone chewing is highly effective in reducing dental calculus. However, the provision of bones carries some risk of oesophageal or intestinal obstruction (55), tooth fracture (56, 57), diarrhoea, or constipation (57). Chews in the brief studies listed above (53, 54) did not cause any of these issues. Notably, all the studies listed involved adult, medium-sized mesocephalic dogs (beagles). In contrast, brachycephalic (58) and tiny breeds (<8 kgs) (55) may be at greater risk of periodontal disease than other sizes and shaped dogs because their extreme morphology compromises physiological masticatory function and, in turn, their ability to chew a substrate safely (58). This phenomenon has been shown in ponies (*n* = 9), where morphology (size and head shape) affects ingestive behaviour and food intake rates when compared with horses (59).

The oral microbiome is associated with dental health and disease. In general terms, gram-positive anaerobes predominate in dental disease, while gram-negative aerobes (commensal) characterise healthy oral states. There is evidence that even short periods of chewing can normalise the oral microbiome and, thus, ultimately reduce dental disease. The same 12 beagles studied above underwent oral microbiota sampling before (Day 0) and after (Day 14) 13 days of chewing autoclaved SB or CB 4 cm bone pieces (60). The SB group showed an increase in commensal bacteria and a reduction in pathogenic bacteria over the 2-week chewing period. The change in these bacterial populations emerged in the SB dogs’ saliva and gingival sulcus samples when compared with their own pre-chewing bacteria profiles. Interestingly, these changes were not seen in saliva or gingival sulcus samples of the CB group, despite calculus removal arising from the provision of both bone types (60). SB was significantly reduced in size or completely eaten, whereas CB had the marrow removed, while the rest remained indented with gnaw notches but were largely uneaten. Complete chewing and dismembering of the SB appeared to have optimised the oral microbiome. Other variables between SB and CB, such as time spent chewing and fat and fibre content, may also have affected the oral microbiome.

##### 3.2.2.2 Digestion

**Figure 1:**
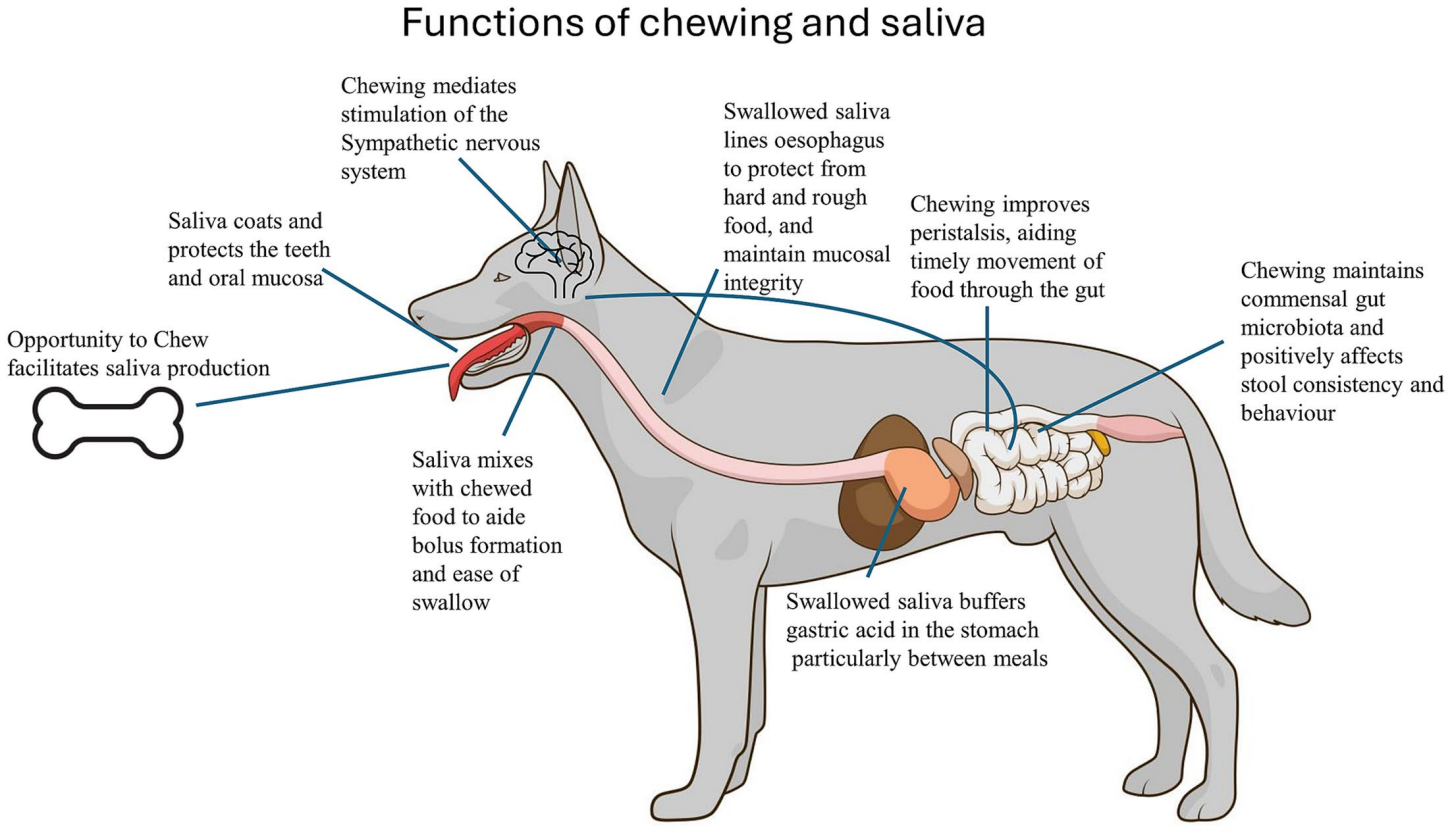
Functions of chewing and saliva production in dogs. A graphic explanation of the various ways chewing and saliva production contribute to digestion, gastrointestinal health, and behaviour.

Chewing aids digestion by effectively increasing the surface area of solid food and increasing exposure to digestive enzymes and saliva in mammals (61). The saliva produced and regularly swallowed in significant volumes may be needed for gastric function and to buffer against gastrointestinal irritation, as shown in horses (62). Dog saliva has a higher calcium concentration and pH (mean pH 7.7) than human saliva (63) and contains bicarbonate ions, all of which buffer gastric acid (64). In humans, chewing increases pharyngeal pH, which may play a role in anti-reflux effects and comfort of the upper digestive tract (65). The viscosity of dog saliva, largely due to mucins, allows it to coat and protect the mucosal and dentine surfaces against injury and bacterial invasion (Figure 1). High viscosity is thought to assist in the rapid deglutition of rough, semi-chewed items, an attribute that increases fitness when conspecific competitors are close by. An association occurs between the eating speed and the activation of opposing arms of the autonomic nervous system. Ohtani et al. (66) found that dogs (*n* = 56) that ate slowly (with chewing) showed peri- and post-feeding activation of the parasympathetic nervous system (PNS), an important stimulator of digestion, and indicated that presenting dogs with foods that require time to chew improves digestion.

Whole foods that contain fibre and that require chewing (compared to powdered diets) optimise the commensal colonic microbiome that is critical in the production of short-chain fatty acids (SCFAs) (67, 68). SCFAs suppress neutrophil recruitment into the colonic mucosa and optimise stool moisture content and transit times, maintain peristalsis, and prevent the extremes of constipation and diarrhoea, as shown in human and animal models (65, 67, 69). In hospitalised humans, chewing gum facilitates the resumption of peristalsis after post-abdominal surgery ileus (70). SCFAs also cross the gut—blood and blood–brain barriers and are implicated in the regulation of cognitive and emotional processes through multiple and complex pathways involved in the bidirectional gut-brain axis (71). Conversely, an imbalance in the gut microbiota is implicated as a factor in anxiety, depression and cognitive disorders in both animal and human models (72).

##### 3.2.2.3 Psychological health

Beyond the GIT, studies of humans, rodents, and dogs indicate that chewing plays a significant role in moderating stress and its various deleterious sequelae. The stress response is an important fitness mechanism in all vertebrates, activated to address and survive potential threats (73). This response involves physiological, behavioural, and psychological activations that generate arousal, motivation, physical readiness, and a behavioural repertoire to help animals confront threats. It represents a transitory and adaptive state, designed to restore homeostasis as quickly as possible, typically within 2 h (74). Homeostasis is maintained by the parasympathetic nervous system (PNS), which predominates during the animal’s rest-and-digest state (75).

**Figure 2:**
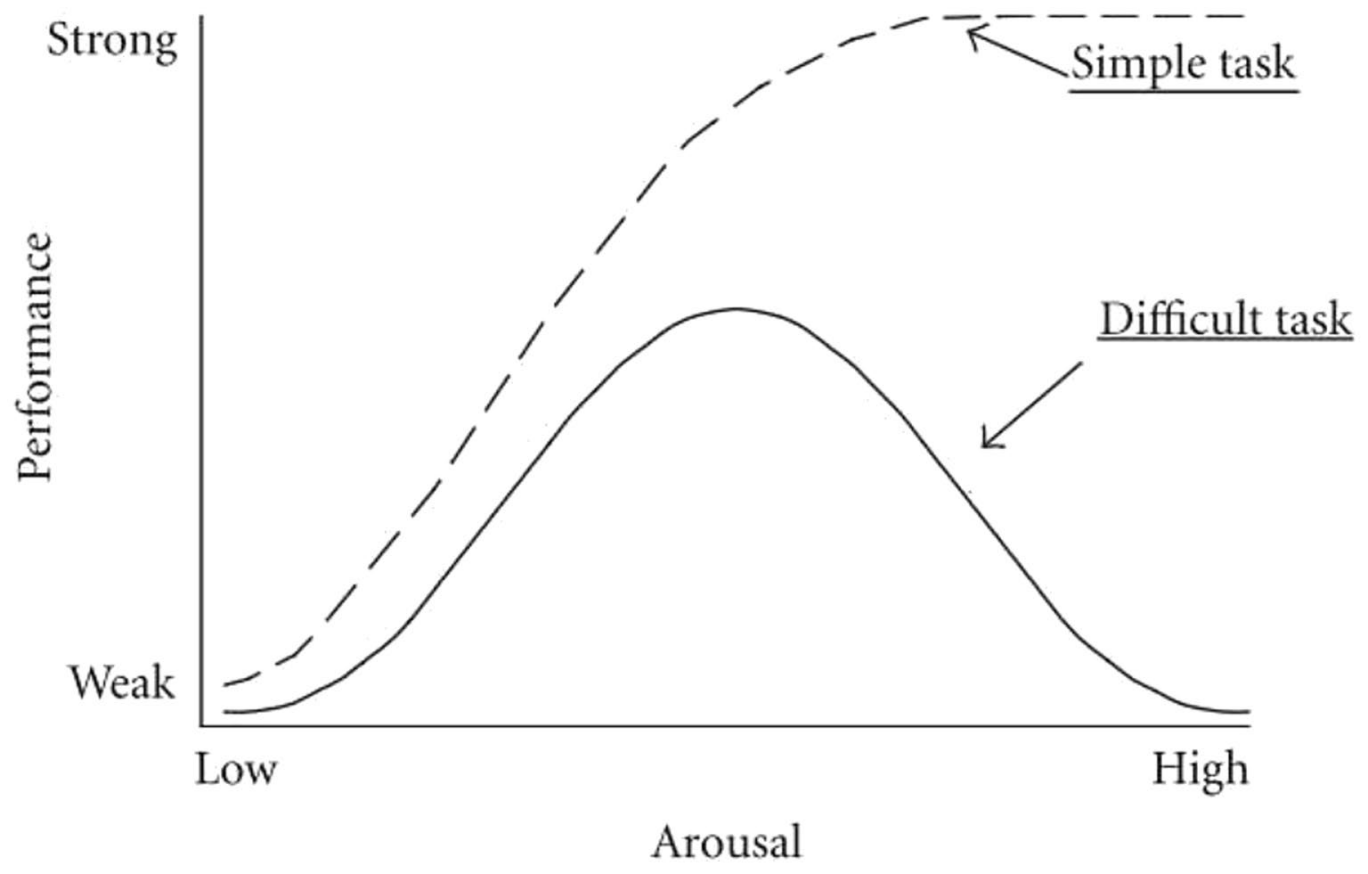
The Yerkes-Dodson law displaying the bell curve link between arousal and performance. Increases in arousal and attention caused by the stress response assist performance (eustress) up to a point, and then increased levels of arousal compromise the ability to learn and perform. Simple tasks, such as singular tasks with a small, known range of cues, may continue to display improved performance at high arousal levels. Source: modified from Diamond et al. (201) and used with permission.

Acute stress may not always be an indicator of negative affect but rather is dependent on the degree of arousal and the individual’s perception of the situation. Yerkes-Dodson (76) first reported that, in mice, a moderate stress response could be beneficial in that it improves learning and performance, thereby improving the ability to manage a triggering challenge (Figure 2) (76). This has been replicated for most simple tasks and difficult tasks when arousal is low. When stressors (something that triggers the stress response) are prolonged, intense or of great difficulty, performance and learning are compromised (2). It has been shown that arousal affects performance in dogs (77) in alignment with the Yerkes-Dodson (YD) law. A complicating factor, as described by Selye (1975), is that individual perception and focus can influence whether a stimulus is identified as a threat or a challenge (78, 79). When a stimulus is perceived as a threat, such as a task beyond the individual’s capability, it causes distress. Conversely, when perceived as a challenge, it captures attention and, if successfully managed, leads to a positive experience known as eustress or “good” stress.

**Figure 3:**
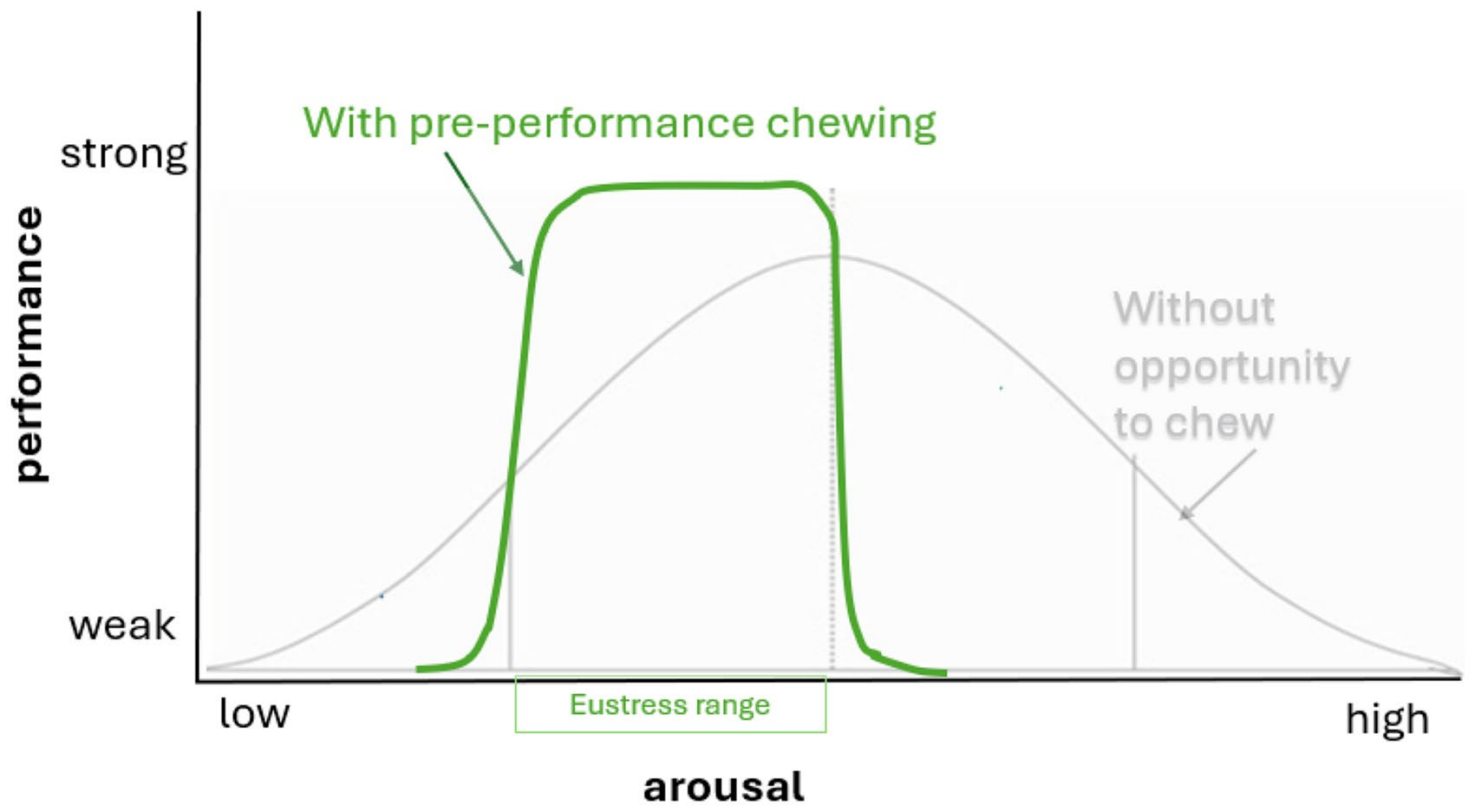
Chewing has been shown to moderate arousal, and allowing dogs the opportunity to chew may help them perform at their best in life situations by promoting or reducing arousal to an optimal (eustress) state (shown in green).

This explains why it is helpful to incorporate the current affective state into stress evaluation of stress. When dogs chew, their arousal may increase or decrease depending on the individual dog’s affective state (80). These theories of acute stress inform our proposal that chewing, as a self-imposed manipulative challenge, may provide the right level of arousal and focus to help dogs maintain an optimal affective state (Figure 3).

Chewing in human and animal-model studies reduces and can ameliorate the negative effects of chronic stress or unregulated/multiple acute stresses occurring over days and weeks (74). Dogs can display acute or chronic distress when separated from their attachment figures (i.e., their dam and later human carers), when deprived of the ability to express normal behaviours, and when placed in novel or threatening situations (81). Chronic stress produces a series of deleterious effects on the body, including hypertension, immune system dysregulation, increased peripheral inflammation (the bladder, the gastrointestinal tract, and the skin), pruritus, inhibition of growth in the young, predisposition to diabetes mellitus, reduction in attention span and memory, predisposition to cognitive deficits later in life, sickness behaviours (suppression of normal behaviour motivation), depression and osteopenia (81–84). Opportunities to chew may reduce the stress of challenging situations as a coping strategy, which has been shown to moderate stress in humans (85), mice (86) and rats (87). This effect occurs because chewing reduces stimulation of the hypothalamic–pituitary–adrenal (HPA) axis and the Sympathetic Nervous System (SNS) (88), which, in turn, improves learning and memory. In human studies (*n* = 40), gum-chewing during stressors (computer multi-tasking tests) increased concentration, reduced self-reported anxiety scores, improved performance [thought to be due to increased alertness (89)], and lowered salivary cortisol concentrations (90, 91).

Within chewing behaviour, chewing intensity and the rate of chewing are key factors that vary to moderate stress levels. This has been shown in humans (*n* = 31), where the rate of food chewing (eating sandwiches and cupcakes) increased by 5–20 min immediately after a stressor (92).

Krichbaum et al. (80) showed links among desexed adult Labrador retrievers between chewing intensity, memory, and cognitive performance. The dogs (*n* = 32) were categorised as fearful or non-fearful using the Canine Behavioural Assessment and Research Questionnaire (C-BARQ). Fearful dogs (number not given) performed better on a spatial memory test immediately after the opportunity to chew a synthetic inedible chew item (Nylabone®) for 5 min. Moreover, those that chewed at a higher bite intensity performed better on a maze test, suggesting that more emphatic chewing improved long-term memory consolidation. These results suggest that chewing may mitigate fearfulness, which would otherwise hinder memory. In contrast, non-fearful dogs (number not given) showed no significant improvement in performance after the opportunity to chew, which aligns with the Bray et al. (77) study, showing that arousal follows the YD bell-curve relationship with performance. These findings indicate that any effects of chewing may depend on the individual’s current emotional state at the onset of chewing and that chewing can either raise or lower arousal. They are significant because they strongly suggest that chewing may affect fitness and highlight the need to assess the affective state in dogs before, during, and after cognition or welfare studies involving chewing interventions.

The properties of the chewed item are another factor in understanding the link between chewing and stress management. In human studies, chewing non-flavoured gum did not reduce cortisol responses, as observed in studies that evaluated the effects of chewing flavoured gum (noting that these studies also differed in that cortisol concentration was tested in plasma vs. saliva) (*n* = 40) (93). Furthermore, sucking gum (that is, holding the flavoured gum in the mouth without chewing) produced some, but not all, of the same benefits for human participants as chewing (*n* = 48) (91). This may highlight the potential for a greater value of food chews in dogs, such as bones and dental chews, due to the contributions of odour and flavour. It may also suggest that, even without ingestion, chewing can have value in terms of mouthfeel and substrate disintegration.

Other examples of the stress-reducing and homeostatic benefits of chewing relate to bone health. Chronic stress in human and animal models (84) or iatrogenic glucocorticoid administration, as shown in Beagles (*n* = 16) (94), can lead to osteopenia because of bone resorption and suppression of bone formation. Azuma et al. (86) reported that adult mice (*n* = 30) exposed to chronic stress over a 4-week period, allowing them to chew on wood, significantly reduced cortisol concentrations, leading to a significant reduction in osteoporosis.

An appropriately functioning masticatory system, which includes chewing, is beneficial to older adults by providing some protection against cognitive decline, as has been shown in aged humans (95) and mice (*n* = 128) (96). Senior mice with previous access to chewable food with their mandibular molars removed developed hippocampal atrophy compared to mice with normal chewing ability (14 days in the non-chew state). This loss of hippocampal structure and function in mice led to cognitive decline in a maze test (97). Senior dogs, defined as those in the last 25% of their anticipated lifespan (based on the average life expectancy for their breed or type) (98), are also likely to show associations between loss of cognitive function and loss of chewing ability due to tooth loss, periodontal disease, and pain that prevents chewing, or simply due to the lack of access to chews. Conversely, in humans, cognitive function appears to be protected by chewing (95).

In summary, chewing appears to benefit numerous body systems. Its primary role is as a consummatory behaviour to physically pre-process food in preparation for swallowing. Additionally, chewing affects all parts of the digestive system, from the oral cavity to the colon. Beyond the gastrointestinal tract, chewing promotes psychological health by moderating the autonomic nervous system and may have prophylactic benefits for cognitive function. Finally, chewing provides protective functions for the skeletal system.

### 3.3 Mechanism

Tinbergen’s third question relates to the mechanical aspects that enable successful chewing throughout a dog’s lifetime. This involves the chewing apparatus, neural control, and bite force, which are common to most dogs and vary with head shape.

The orientation of an item for chewing involves using the forepaws, which have been studied as part of canine motor laterality (99). The paw placed on top of the focal object is lateralised in many individuals and some populations (100). Laterality is reduced after acute and chronic stress in dogs (101), and paw use while chewing may be useful as an indicator of stress in individuals with known paw preferences. The item is secured to present it for optimal chewing, depending on its shape, type, and surface characteristics. Dogs’ wide gape and mobile jowls allow the capture of prey or the carriage of other large items, such as coconuts, and lateral manipulation of large food items toward their cutting and crushing teeth (19). For example, when stripping the periosteum from a bone, a dog may orient the bone horizontally, hold it with their paws, and pull with their incisors. In contrast, when cracking a bone, the dog may hold it vertically to optimise access for the molars, as described in wild canids (*Lycaon pictus*) (2). These manipulative skills may not be consistent across all morphotypes (99). Brachycephalic dogs exhibit longer latency when opening food puzzles, use their paws less, and rely more on their carers than mesocephalic dogs (102).

**Figure 4:**
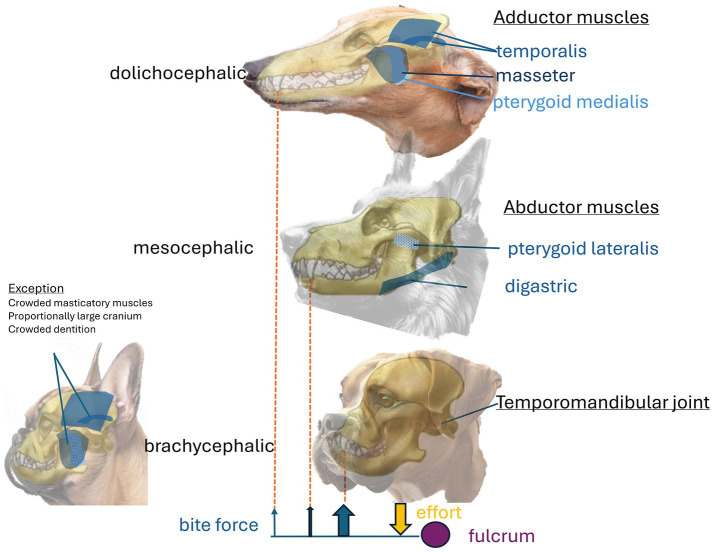
illustrates the major masticatory muscles and bite force schematics for each skull shape. The adductor muscles close the jaw and generate bite force. The primary adductors are the masseter and temporalis muscles on the lateral sides, along with the pterygoid medialis muscle situated deep within the masseter. The abductor muscles open the mouth, including the digastric and pterygoid lateralis muscles, which lie and attach rostromedially to the mandible. Bite force at the canine depends on head shape. A longer lever arm (the mandible) results in muscles applying effort further from the fulcrum (temporomandibular joint). Consequently, the dolichocephalic skull shape generates the least bite force, while the brachycephalic skull exhibits the most, relative to mass. An exception exists among small-breed brachycephalic dogs, which demonstrate less bite force than small-breed mesocephalic dogs, known for the strongest bite force. This discrepancy is likely multifactorial, stemming from the crowding of mastication muscles on the proportionally larger cranium and crowded dentition.

In mammals, chewing involves complex, rhythmic mandibular movements that are semi-autonomous. These movements are regulated by regions of the brainstem, which receive input from other areas of the brain involved in the integration and processing of the oral sensory inputs. Chewing engages a significant proportion of the head and jaw muscles (103). In dogs, chewing is secodont (scissor-like), occurring in the vertical plane without side-to-side movements, which enhances cutting and crushing abilities (26). The jaw closes through the contraction of strong adductor muscles, particularly the masseter, temporalis, and pterygoid medialis muscles (Figure 4). Adduction generates the bite force applied over the occlusal surface during chewing. Reflex and isotonic co-contraction of the abductors, specifically the pterygoid lateralis and digastric muscles, along with a pause in adductor muscle activity, result in a rapid decrease in velocity and a corresponding reduction in force that protects the dentition and the mouth during chewing (104, 105).

The bite force is consistently greater at the carnassial tooth than at the canine (106) due to its proximity to the muscles and the temporo-mandibular joint (19). Estimates based on dry skull models and allometries of various canids, felids, and ursids indicate that within a species, bite force is related to mass and cephalic index (skull width × 100/skull length). For example, the grey wolf (*Canis lupus*), with an average body mass of 55 kg, exhibits a maximal bite force of 1262.3 N at the carnassial tooth and 743.0 N at the canine. In comparison, the maned wolf (*Chrysocyon brachyurus*), with an average body mass of 23 kg, shows a maximal bite force of 725.3 N at the carnassial tooth and 435.6 N at the canine (*n* = 56) (107). In dogs, bite force is further influenced by head shape, with brachycephalic dogs generating the highest force and dolichocephalic dogs the lowest. Brachycephaly may have provided an advantage for dogs historically bred for bull-baiting and dog fighting, as the shortened nose reduces exposure to injury by making it less protruding and harder to reach for a kick or a bite.

Additionally, brachycephalic dogs may exhibit enhanced bite performance due to an increased bite force. Conversely, the long mandibular ramus in the dolichocephalic shape, which forms a longer lever arm, produces less bite force (26). However, this relationship between head shape and bite force does not hold in small breeds (<13 cm skull length), where the mesocephalic shape has the largest bite force, likely (106) due to the large skull allowing less space for masseter muscles and thus affecting their bite ability (26).

Mammals’ masticatory rhythms (chew counts or cycles as a function of time) are largely fixed, both at a species and an individual level, irrespective of the substrate being chewed. Chew cycles are modulated through the masticatory central pattern generator network in the brainstem as part of the autonomic nervous system (104, 108). Thus, the chew rhythm is largely set, while the number of chew cycles and bite force vary depending on the chewed substrate. Masticatory rhythms scale allometrically with mass in mammals (chew count $=aM^{1/4}to\;aM^{1/3})$ . For example, a spiny mouse (*Acomys dimidiatus*) weighing 50 g has a chewing cycle of 282 ms, which equates to a chew rate of 300 chews/min, whereas an African elephant (*Loxodonta africana*) weighing 2,812,273 g has a chewing cycle of 1,530 ms equivalent to 39.2 chews/min (109).

In contrast, compared to size-matched non-domestic mammalian species, dogs (*Canis familiaris*) only show a small correlation between chew rate and mass (*n* = 4/breed for 31 breeds) (108). This may represent a loss of physical and biological fitness due to the rapid change in morphology from artificial breeding for extremes over an evolutionarily brief period (110). Examples include large dogs with a relatively fast chew rate for their mass and overrepresented with tooth fractures (111). Small dogs that have a relatively slow chew rate based on mass may be overrepresented with obstructive conditions (55). Alternatively, dogs of all sizes may still be meeting their fitness requirements because there is less selection pressure for efficient chewing due to anthropogenic diets. Note that others found that the mandible and muscles of mastication were modified together and appropriately for the requirements of chewing, irrespective of size and morphotype (*n* = 48) (112).

Both tiny and brachycephalic breeds share factors that hinder natural chewing and increase the risk of adverse dental consequences. Tiny dogs have large teeth in relation to their mandibular bone, which may be significant because tooth roots can penetrate deep into the ventral cortex of the mandibular bone. This bone-tooth ratio reduces tooth stability and increases the likelihood of mandibular fractures, even from relatively minor forces (58). Additional factors that contribute to a higher risk of dental injury include dental crowding, tooth rotations, malocclusion, traumatic buccal granulomas, under-erupted teeth, and a loose mandibular symphysis (58). These factors are characteristic of brachycephalic breeds (notably Boxers, Boston terriers, pugs, French bulldogs, and Shih Tzus) and particularly diminutive individuals within those breeds (58). These factors are important to consider when evaluating the properties of chews for various dog breeds.

Canine teeth in canids have evolved to withstand high rostral-caudal forces, which occur during the capture of moving prey (113). However, these canines are not as resistant to lateral (lingual to buccal) forces, and this mismatch may explain some of the fractures reported in canine teeth (113), along with a lack of chewing experience to strengthen the chewing apparatus, discrepancies in chewing rhythm, and perhaps a risk associated with the dog’s condition.

The qualities of the substrate affect what dogs choose to chew. Olfaction is a dog’s primary sense, accounting for a volume of the brain that is 30 times larger proportionally than in humans (114), which plays a significant role in sourcing food. Furthermore, taste (115) and texture (or mouthfeel) determine consumption preferences; dogs that initially select food based on smell will decrease their intake of that food over time if the substrate does not match the odour (116). The first bite assesses the hardness of the substrate, and when measured in humans, it is not at full force (117). Pressoreceptors in the periodontal ligament, mechanoreceptors in the masseter muscles, and sensations in the mouth provide further feedback to inform bite force and the number of chew bouts (104).

Comparative chew studies covering a range of dog morphotypes would be valuable. Dogs are mechanically well-constructed to manage a range of chews, including large and hard items.

### 3.4 Ontogeny

Finally, it is important to understand how chewing behaviour develops throughout an individual’s life. Chewing affects the neonate in utero, during development, through the challenges of life, and as an animal ages.

#### 3.4.1 Maturation and growth

In the ethogram of sub-adult dogs (puppies, juveniles, and adolescents up to 30 months old, depending on the size or type), chewing is essential for normal development and maturation (81, 118). The onset of weaning and the transition to solid food coincide with a series of developmentally connected mechanisms. The eruption of deciduous teeth and feedback from periodontal mechanoreceptors trigger the maturation of the masticatory centre and the beginning of a chewing rhythm (*n* = 50) (118). Together, the neural reflex, sensory feedback from the oral cavity, and the development of adductor muscles enable pups to mechanically break down food through chewing. This aligns with the puppy’s transitional period (2–3 weeks old). The opportunity to chew ensures that the masticatory apparatus becomes experienced and capable of managing a variety of food items. The modern approach of providing weaning pups with soft, mushy puppy food—either wetted dry food or tinned puppy food—may hinder chewing patterns and muscle development, potentially impacting jaw and overall facial strength and development, as observed in human studies (119). In free-ranging settings, and perhaps in captive environments as well, dogs have the option to bring back parts of a carcass or other whole foods for the puppies to chew and play with (120). These items may help the pups when the dam begins to leave the nest for extended periods at 2–3 weeks postpartum. Wild dogs have been observed sucking, licking, chewing, and playing with these items, mostly (74% of the time observed) in the absence of an adult dog (28). These behaviours indicate a motivation to interact and exhibit a positive effect, possibly providing a positive distraction, abatement from hunger, and helping to keep the pups within the area around the den, where they are relatively hidden from predators.

The growth and movement of permanent teeth under the gums and their subsequent eruption during the teething process (from 4 to 30 weeks old) are believed to motivate puppies to chew in order to alleviate discomfort. However, the increased motivation to chew may not be as closely associated with discomfort (as noted in human literature) (121) and, in dogs, is more likely linked to the activation of the masticatory centre and the processes of development, maturation, and weaning (118). The sensory change brought about by erupting teeth in the mouth triggers the maturation of the masticatory centre in the brainstem, initiating an autonomic and rhythmic chewing pattern that aids in the ingestion of semi-solid food. Early usage of the chewing apparatus highlights the importance of tissues engaging in the work expected of them in adulthood. Just as bone is reinforced by mechanical loading (122), the periodontal ligaments and jaw bones strengthen in response to the pressures and tensile forces that develop with maturation and chewing (52, 119, 123, 124).

Young dogs also chew as part of play. Bradshaw et al. (4) suggest that play functions facilitate the development of adult predatory, agonistic, and ingestive behaviours. These behaviours rely, in part, on the muscle strength, endurance, and dexterity of the chewing apparatus. Normal development of chewing occurs without the need to play with targeted objects. However, play such as jaw sparring in play-fighting and object play improves adult motor skills (125, 126). Animals with less experience playing with chews may develop less nuanced chewing skills, and therefore, be less efficient at acquiring nourishment from them. Dogs are believed to play into adulthood more than most other species (4). This highlights the persistent role of object play in adult dogs, varying depending on object type (125). This suggests that the availability of objects that can be mouthed and chewed may influence their usefulness for play. Some dogs may engage only with items that are also food, such as throwing a bone and then chewing it. Others pursue only those items that can be dismembered in ways akin to predatory behaviour. However, it is acknowledged that the rate of play behaviour varies among individual adult dogs, with some playing minimally or not at all. Play reduces the fear of novel situations (127), and it can be suggested that dogs, when given the opportunity to play with chews, are more adventurous, less fearful, and better equipped to handle new chew items than those without such experiences.

#### 3.4.2 Adulthood

Agonistic behaviour is mostly demonstrative and typically resolved without injury (81, 128). Additionally, practice with chew items may contribute to conditioning for adult life (129). Access to plentiful chew items is likely to reduce the prevalence of protective behaviour, which may occur among pups when only one or two items are available. Limited resources tend to hold more value than abundant ones, as described below in *interactions with other animals*.

Whole food items brought to the nest cannot be quickly ingested and may draw the attention of pups while also providing a focus for their social behaviour development. Wild dingo pups in Australia were observed spending extended periods around a carcass (28). This may have occurred during a time when the expression of play-fighting was beginning to emerge in these pups (beginning at approximately 5 weeks old, peaking at 8–9 weeks in free-ranging dogs) (130). Such early agonistic behaviour may play a role in socialisation (131) and have a significant impact on learning in social species such as canids. Chews may stimulate social learning.

Neophobia and neophilia can impact the choice of chews. A tendency toward neophobia (the avoidance of novelty), combined with an early preference for familiar foods (the primacy effect) (132), may lead dogs to avoid novel food items, especially when they are first introduced to them (133). Neophobia observed in dogs, cats (1), rats (134), and birds (135) may also extend to chew items in fearful or stressed dogs. Conversely, a novelty effect, which involves preferring a new food over one regularly fed (1, 132, 136), can occur in dogs and other species. Deprivation and malnutrition in early life may render juvenile animals capricious in their food choices (137).

It has yet to be determined whether dogs with limited access to bones, and therefore little experience with them (and possibly the mineral concentrations necessary for bone and tooth strength), are more vulnerable to such risks. Insufficient chewing has been linked to the loss of masticatory alveolar bone in dogs (*n* = 1,350) (42) and in rats (*n* = 60) (138). In addition to the characteristics of the dogs, the hardness, smoothness, and low dissolvability of the chewed item were risk factors for it becoming lodged in the gastrointestinal tract (especially in the distal oesophagus) (55). This lodging can lead to tissue necrosis of the alimentary tract wall, complicating the surgical extraction of the chew. Additionally, the shape of certain chews may encourage premature swallowing (55). There may be co-development between the functionality of the dog’s digestive tract and the irregularities of natural items.

Companion dogs sometimes chew on non-nutritive items, such as sticks and toys, that can be stripped and broken down, with the pieces being spat out. Companion dog owners often actively encourage oral interactions with non-nutritive items, including ropes, rubber toys, plush toys, cardboard, and balls. This practice is driven by the pet merchandise industry, anthropomorphic ideals, and guardians’ beliefs that chewing opportunities are important (139), enjoyable (140) or enriching (141) for their pets. Indeed, the apparent surge in demand at pet supply stores suggests that owners are recognising their dogs’ need for such oral forms of enrichment in addition to the provision of food.

However, prolonged gnawing on non-nutritive items, such as tennis balls or even on themselves due to chronic pruritus, can cause significant wear to the incisor and canine teeth in particular, expose pulp cavities, and lead to non-vital teeth that require extraction (142). Swallowing non-nutritive items, referred to as pica (143), is believed to have a multifactorial aetiology, including obsessive-compulsive disorder, anxiety, impulse control issues, and nutritional deficiencies (144), or may occur as a protective behaviour. In some cases, particularly in juveniles, providing nutritious, strip-able chews may help redirect motivation away from non-nutritive items.

Learning deficits in offspring born to a stressed dam may be improved by allowing the dam to chew. In mouse models, dams experiencing stress can have their stress response alleviated by the opportunity to chew. This, in turn, prevents the reductions in neurogenesis in the hippocampus of developing foetuses induced by adrenal corticosteroids (*n* = 24 mice) (145).

A dog’s bite force and chewing ability can be compromised by oral or jaw pain, which is most often caused by periodontitis and osteoarthritic pain in the temporomandibular joint (26). Pain becomes particularly relevant as dogs age, yet the importance of chewing continues, as it helps maintain cognitive function and protects against cognitive dysfunction (95).

Ontogenically, the events that an individual canid faces throughout its life may be modulated by that dog’s access to chewing substrates. As documented in wild canids and supported by research across species, dogs are built to chew and are motivated to do so from the teething stage to old age.

**Figure 5:**
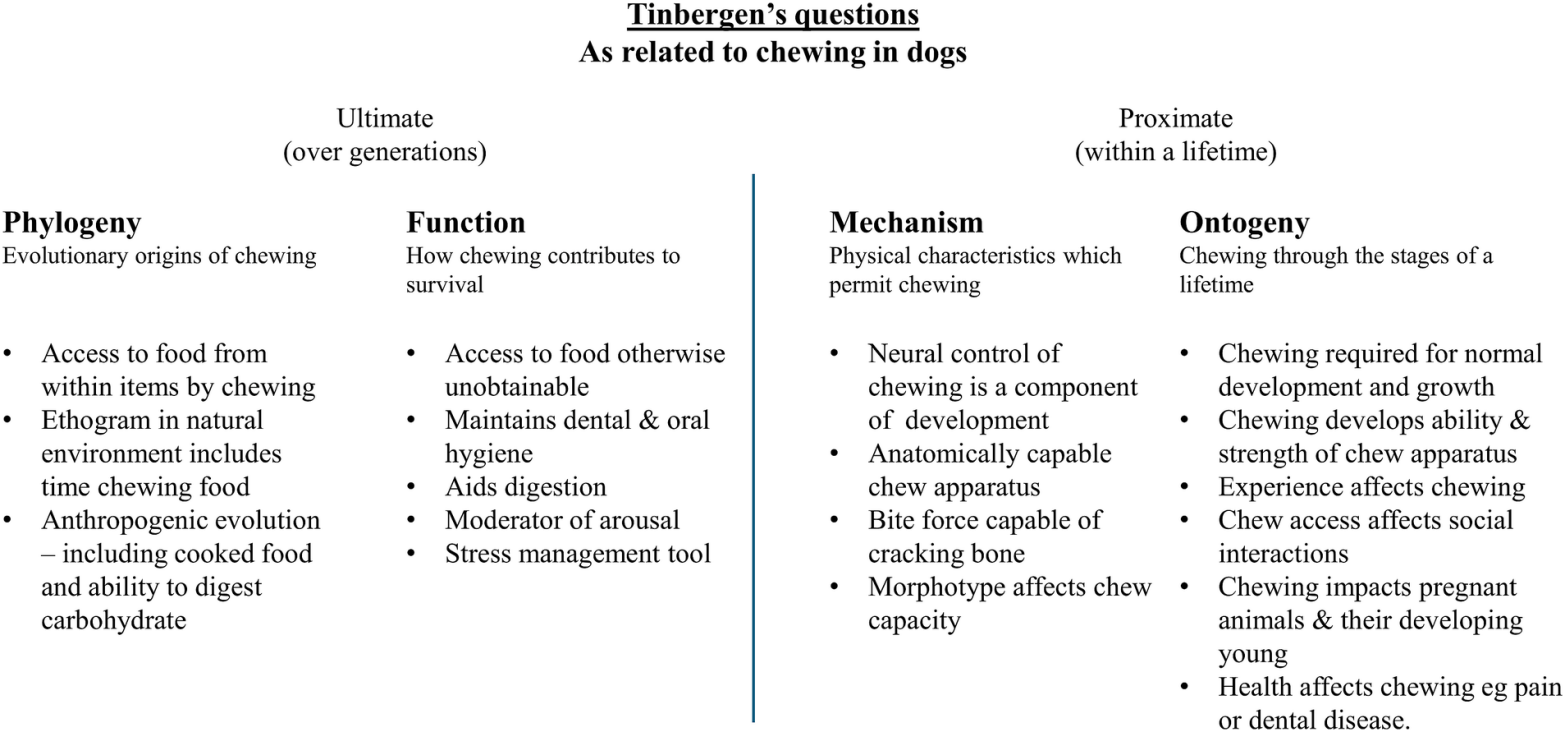
A summary of Tinbergen’s questions as they related to chewing in dogs.

In summary, chewing plays a role in all four of Tinbergen’s questions, as outlined in Figure 5. Ultimately, chewing has influenced and is influenced by the dog’s evolution (phylogeny) and fitness (function). Meanwhile, dogs are mechanically capable and chewing impacts how they live in all life stages.

## 4 The five domains

**Figure 6:**
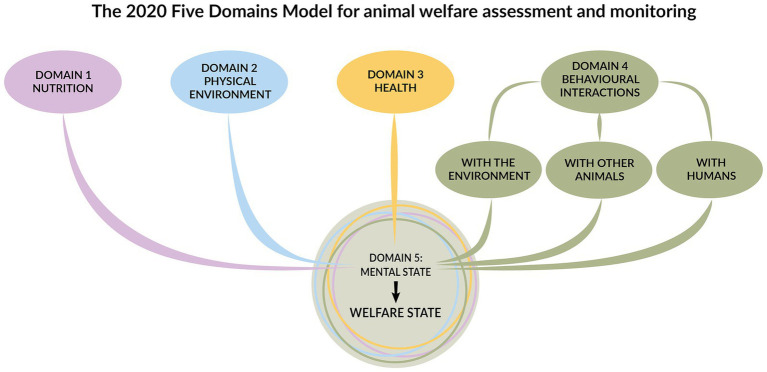
The five domains model for animal welfare assessment and monitoring includes the four domains: nutrition, physical environment, health, and behavioural interactions, which collectively influence the fifth domain, representing the animal’s welfare state. Adapted from Mellor et al. (6) by Cristina Wilkins and used with permission.

Formulated in 1994 (146) and most recently updated in 2020 (6), the Five Domains Model is an animal welfare assessment framework. Initially established for sentient animals in research, teaching, or testing procedures, it is now more widely valued for any animal(s) with which humans interact. The model recognises the dynamic link between biological function and affective state, as well as the importance of assessing both the negative and positive impacts of human behaviour on animal welfare across the four physical domains: nutrition, physical environment, health, and behavioural interactions. The framework provides an index of how each domain contributes to the animal’s mental state (i.e., the fifth domain) and the animal’s overall welfare state (6) (Figure 6). In this study, we used the model to evaluate chewing opportunities in dogs.

### 4.1 Nutrition

This commentary assumes that the dogs in question are meeting their nutritional needs with current rations. However, in nature, it is possible that some nourishment needed for primary nutrition is sequestered within hard or fibrous items, such as bones, nuts, or seeds, and may become available only through chewing. Anticipatory behaviour and goal-directed exploration toward such sequestered food can bring satisfaction and pleasure that do not arise with the freely provided food (140, 147). Additionally, chewing is one method to slow food ingestion rate, improving digestion (66) and nutrition.

Dogs, even when sated, display positive anticipation (148), focused attention, and positive body language (149) as indicators of positive affective state (6). Positive body language includes horizontal, right-sided tail-wagging (150), active movement toward and sniffing the item, soft muscle tone, relaxed eyelids, ears positioned neutrally on the side of the head or forward, and an open mouth (unless sniffing, during which the mouth closes) (81). Positive affect arises from dopaminergic pathways of reward during food acquisition and positive consummatory reinforcement. These pathways may play a role in the animal’s overall assertiveness (151) or confidence and willingness to seek items or environments they value. This is observed when dogs engage and persist in solving familiar tasks (152). Conversely, a lack of ability to achieve the goal and consume food concealed within items, such as food in complex puzzles or marrow in bones, may cause frustration or depression (i.e., negative states). This behaviour may be observed, for example, when morphology, pain, dental disease, or health issues compromise a dog’s capacity.

Chewing adds to the diversity of a diet, as each mechanical interaction with a solid chew is unique and alters the shape, texture, and taste of the target, particularly natural items like bones or plant materials. Flavour is released when confined food becomes accessible from chewing and mixing with saliva. Dogs that are given a processed and homogenised diet may become frustrated (and perhaps bored) due to a lack of both variety and opportunities to engage in appetitive behaviours (140, 151). Therefore, chewing may play a crucial role in alleviating these potential deficits.

Chewing and the food extraction over time may be a form of contra-freeloading. In ethology, contra-freeloading is the phenomenon whereby animals that have been offered both a free-access food source and one need to apply effort to choose the food that requires effort (153, 154). Wild canids exhibit contra-freeloading, likely because foraging is an essential appetitive behaviour for survival in these species, serving as a rewarding fitness criterion even before consumption. Dogs are demonstrably willing to contra-freeload but often prefer free-feeding when given the choice, such as when using a snuffle mat (a tufted area of fabric that requires the dog to sniff and manipulate the material to access the food) (*n* = 38) (155). This preference is also seen in domestic settings where companion cats and dogs favour free feeding. Companion animals typically obtain food from humans and are generally not required to forage for survival. Due to prolonged artificial selection, they may have lost some problem-solving abilities (and motivation) regarding food in a trade-off for an enhanced ability to interpret human social cues for food access (156). While contra-freeloading is characteristic of non-domestic canids, companion dogs willingly engage in it and naturally partake in foraging behaviours (154), highlighting a potential deficit in the domestic environment. The absence of contra-freeloading may compromise welfare if the lack of species-specific and naturally challenging food acquisition (157) leads to negative affective states such as frustration.

Overall, while adequate nutrition is often achievable with minimal chewing, adding chewing to food ingestion offers an opportunity to improve welfare.

### 4.2 Physical environment

The physical environment encompasses the domain related to the thermal and structural properties of the animal’s surroundings and how these attributes affect welfare (6). Focusing on chewing, this domain affects a dog’s ability to engage in this activity when conditions are sub-optimal. We provide examples of suboptimal physical conditions and briefly consider their influence on chewing. For example, given that the mouth is needed for both panting and chewing when the ambient temperature is outside the dog’s thermoneutral zone, thermoregulation may be more important than chewing. Furthermore, the confined environment of kennelled (158, 159) and many companion dogs (160) may restrict access to resources and limit opportunities to express species-specific behaviours (161, 162), including chewing. This restriction may compromise welfare.

Dogs may prefer to chew under shelter rather than be directly exposed to inclement conditions. However, companion dogs housed outdoors might have greater access to fresh, meaty, fatty chews than those kept indoors. Additionally, some pliable ground surfaces may be more suitable for chewing than harder or shinier ones, which can frustrate the dog’s attempts to maintain a steady chew while chewing.

It appears likely that dogs evolved to be surrounded by debris from carcasses in their dens; thus, their typical living environment is characterized by the odours that may arise from such debris. Dogs have the ability to influence their own environment over time, particularly with chewable items and various remnants from carcasses, which may lead to positive welfare outcomes (6). However, when new dens cannot be easily found in confined environments, it may also attract scavengers and allow pathogens to proliferate. This domain and the behavioural interactions with the environment overlap, prompting considerations of chewing as it contributes to and is influenced by the dog’s living space, as discussed in Domain 4.a. behavioural interactions with the environment. It is suggested that chewing opportunities in a dog’s environment may provide a pathway to positive welfare.

### 4.3 Health and fitness

Chewing requires the physical engagement of various muscles in the head, neck, and forepaws, thereby necessitating energy expenditure and cognitive processing to obtain sequestered nutrients. This process may increase the tendency for post-chew rest and sleep, similar to what is observed after exercise in humans (163). Sleep is critical for welfare as it is an essential behaviour with a strong rebound effect when inhibited (164). It plays a vital role in energy conservation, protection during dangerous times (especially for the young and old that need to evade predators), immune stabilisation, consolidation of memory and other brain functions, and recovery from oxidative stress (165).

Chewing improves fitness by benefitting oral and dental hygiene, psychological health, and bone health. It serves as low-intensity physical exercise, a stress amelioration and prophylaxis tool, as described in human and animal models (166, 167). Chewing has a positive effect because it aligns with canine telos, can be conducted in relatively safe spaces, and may occur in the absence of direct threats. Consequently, it may become a comfort behaviour not just for neutral welfare but also for enhanced welfare, primarily indulged in when the stomach is relatively full.

There are potential negative health outcomes from chews, some of which may arise from inadequate chewing options, especially for puppies and juveniles. This is when chewing on other objects is reported most commonly (139) and when socialisation and experience (168) can affect future willingness to engage in chewing as well as the skill to chew effectively. These outcomes have yet to be tested empirically. Failure to provide adequate chewing options may compromise the strength or dexterity of the chewing apparatus or cause dogs to become too excited when they finally encounter a chewable item to chew safely. If a broken tooth acutely exposes the pulp cavity, this can lead to pain through nerve exposure. Broken teeth can also change the process and outcomes of chewing, subsequently impacting the success of future food acquisition and digestion. When an item has been poorly chewed, it can become lodged in the lumen of the intestine, potentially resulting in a fatal outcome without surgical intervention. The accumulation and dehydration of chews in the large intestine can lead to constipation. Some materials, such as cooked bones, pose a particularly high risk, partly because they create significant friction against the intestinal lining. Certain items may inflame or otherwise irritate the gastrointestinal tract (GIT) and lead to cramping, gas production, pain, diarrhoea, and inflammation that can make the GIT vulnerable to ulceration, haemorrhage, and infection. However, these are uncommon sequelae that can occur with various substrates, not only chews.

Lip licking, when observed separately from chewing and eating, is recognised as a displacement behaviour in dogs (81) and occurs in humans experiencing psychological stress (169). Displacement behaviours are normal behaviours displayed out of context and are believed to help animals cope with minor stressors or internal conflicts. Considering the function of canine lip licking as a displacement during arousal, lip licking and concurrent salivation may attempt to alleviate a dry mouth, which results from the SNS’s stress response.

In summary, a dog’s health and fitness can be positively or negatively impacted by the substrates provided for chewing. Carers should choose chewing options that align with the dog’s morphotype and age. An informed approach to chew selection risk management increases the likelihood of positive health outcomes and good welfare.

### 4.4 Behavioural interactions

Dogs form relationships with other animals, humans, and (beyond thermal and physical comfort, see Domain 2 above) the environment they inhabit, all of which influence their behaviour. Carers are the most influential humans for a companion dog, and how they interact with chews can significantly impact the dog’s experience with them. Dogs engage with other animals as predators of prey, as conspecifics, and as social affiliates. When considered in the context of chewing, these relationships can be natural and positive or unnatural and negative. These aspects are discussed here.

#### 4.4.1 Interactions with the environment

An environment rich in chewing options can provide significant enrichment. Allowing dogs *ad libitum* access to chewing enables them to interact when and where they wish. Dogs that can effectively problem-solve and manipulate their chewing to obtain food may experience a particularly positive affective state as a result (170). These choices likely enhance the value of the item compared to food in a bowl, which presents a negligible cognitive challenge. Chews that vary (whether because each bone is inherently unique or represents various chewable substrates) provide enrichment through the exploration and stimulation that novelty brings. Chewing may be a behaviour that dogs indulge in when they feel relatively safe and satisfied. Therefore, the affective state of dogs while chewing may contribute to their perception of safety in the current environment.

How an individual dog interacts with chew and its ability to persevere with complex tasks reflects and influences its affective state. Positive states may allow dogs to be optimistic and appreciate that food is within a hidden source (170), thus motivating them to persevere and find it (8). Furthermore, interacting with and finding food will likely evoke positive emotions through reward pathways (170).

An environment rich in opportunities for chewing can provide significant sensory stimulation for dogs. Given that a dog’s primary sense is smell, the scent of a chew has a substantial impact on the dog’s interest in the item and its decision to lick and mouth it. The motivation to chew is determined by both smell and taste, assessed mainly through receptors in the tongue and the texture of the item in their mouth, including its hardness. Chewing benefits from positive associations formed through previous experiences (91) and promotes playful interactions, often referred to as object play. Additionally, chewing can serve as a positive distraction for dogs adjusting to separation from their group members and attachment figures (171). Indeed, chews may be more accessible when competition from conspecifics is absent.

When nutrition does not depend on it, chewing can be considered a form of play. Play is defined as a broad range of voluntary, truncated behaviour patterns that incur cognitive and physical energy costs and accompany a positive affective state (4). It is affected by stressors such as overcrowding and extreme ambient temperatures. Play is most prevalent in animals that are relaxed and free from threats, discomfort, hunger, danger, and illness (126). Chewing may be a component of play and, therefore, could serve as a proxy indicator of appropriate environmental enrichment and an overall positive welfare state.

Survival criteria are less critical for companion animals than for those in the wild; therefore, their behaviour may not be as strongly constrained by the need for biological fitness (172). Thus, the domestic context may allow more time for play. Play may occur more frequently in companion, zoo, and laboratory canids with a sufficient and reliable food source than in free-ranging and wild canids, filling potential vacuum periods. Vacuum periods refer to times in a confined animal’s day that would otherwise be occupied by fitness behaviours such as foraging, hunting, and chewing for nourishment. When such fitness behaviours are no longer necessary, dogs may still be motivated to engage in analogous behaviours, such as chewing, licking themselves, or dismembering toys.

Chewing may fulfil appetitive motivations, while the denial of the opportunity to chew appropriate substrates may leave these motivations unfulfiled and cause frustration (173). To achieve oral satisfaction, dogs seeking to fulfil their need to chew may target available but inappropriate substrates or those valued by humans, such as bedding or furniture. Indeed, these items can cause dental erosion if chronically chewed and damage the gastrointestinal tract if swallowed (174) or may be viewed by caregivers as part of destructive behaviour when directed toward inappropriate items. Furthermore, unmet internal motivations may lead to stereotypic behaviours (175), so we may propose that a lack of chewing may be a risk factor for oral stereotypies, destructive chewing, excessive barking, or repetitive grooming/licking.

Veterinarians in Spain (*n* = 236) reported that the most frequent behaviour-related complaint from owners was destructiveness (176). Similarly, a study of dogs relinquished to an Australian shelter found that of the 11% of all dogs relinquished for behaviour-related reasons, 6.8% were due to destructive behaviour. Destructive chewing is most prevalent in dogs under 1 year of age (139), implying, as discussed elsewhere, that this age group requires more opportunity to chew than others and that this predisposition may be entirely normal (81). Destructive chewing in all age groups is likely to be accompanied by certain environmental triggers, such as being home alone (177) or a change in routine (139), indicating that destructive chewing may be an attempt to reduce arousal (131). Destructive chewing in these instances is a symptom, both assisting the animal in managing or moderating a stressor and providing a red flag for carers who need to consider the dog’s emotional state and the likely need to enrich their environment. This may include the provision of appropriate freedom to chew.

Normal ethograms may depend on access to chewing behaviour. DeLuca and Kranda (178), studying laboratory dogs, observed that the value of an enrichment item was determined by how much it could be chewed. The provision of chew toy enrichment in the form of a non-chewable latex cylinder (Kong™) filled with food, along with daily positive reinforcement sessions that rewarded desirable behaviours, improves the proportion of desirable behaviours displayed by shelter dogs (*n* = 58) (179). Further research to evaluate behaviour performed after the provision of chewing would be valuable in establishing how well anthropocentric “desirable behaviours” align with positive affect in dogs.

If the physical environment is suitable, dogs will cache food for later, taking time to choose a suitable location to place or bury the item. Wild canids carry the item in their mouth (180) to a concealed spot, often using a pre-existing shallow divot in the ground and favouring areas close to the den (181), where they then dig with their forepaws. This may explain why indoor dogs appear to attempt caching in the depression of a sofa or under a pillow. Dogs exhibit ritualistic, instinctive burying behaviours, scooping with the bridge of the nose and tamping with the nasal planum to cover the item (182). Caching behaviour may also be observed in dogs that are already sated with food. Thus, caching may not only be typical but also calming for dogs. It also allows the dog the agency to unearth the item and resume chewing at will. An increase in agency and choice is likely associated with positive welfare (183).

Conversely, if cached or discarded chew remnants are removed by human carers or by conspecifics, this and lack of control may induce body language of frustration (184) and stress for the dog and add to a negative emotional state. A lack of choice to chew or being thwarted from attempts to chew are potential stressors. Other examples may be food presented only once in 24 h. Free-ranging dogs usually eat 4–8 meals per day (132), which may indicate availability and motivation and suggest what companion dogs would prefer if they had more frequent access to food. This pattern may also reflect the needs arising from scarcity and hunger. Rashotte et al. (185) found that kennelled dogs consistently ate at dawn and at random times throughout the day. Since dogs likely eat when food is available, this diurnal behaviour may reflect food availability, such as that of crepuscular prey.

#### 4.4.2 Interactions with other animals

Dogs in groups are more likely to adopt an eat-first-evaluate-later strategy, while individual dogs tend to sample more thoroughly by sniffing and licking before consuming (20). Some competition and agonistic behaviour among conspecifics may be a normal part of a dog’s repertoire, as they are a social species where outright aggression is rare (186). However, the approach of others (including humans) toward valued chews, along with the lack of space to move away, can be distressing. Agonism may be alleviated or minimised by ensuring that the resource is abundant, thereby reducing competition. If serious conflict or protective aggression occurs in multi-dog environments, chews that can be completely consumed in one sitting may be preferable to large bones or other long-lasting chews.

Chews can cause conflicts with other dogs, animals, and humans because their value makes them worth protecting. This highlights the importance of chewing beyond mere nutrition. Interactions with other dogs in the home can become agonistic if there is a lot of protective and heightened behaviour around chews. The motivation to gain access to another dog’s chew may also be high, increasing the risk of arousal, agonistic behaviour, and possibly a physical fight between conspecifics. Fights can compromise social bonds and impact welfare due to the possibility of injuries and, for one dog, the loss of a valued item and the future pleasure of chewing it. In competitive conditions, some dogs may become hypervigilant to protect resources, which is an aroused state associated with negative affect. Additionally, they may be predisposed to less chewing and premature swallowing. This lack of experience with chews may increase the risk of negative sequelae; for instance, injudiciously gulping semi-chewed substrates may elevate the risk of gastrointestinal obstructions.

If resources are not limited (and depending on the previous experiences and relationships of the dogs), agonistic behaviour can facilitate the resolution of temporary conflicts. Dogs possess an extensive range of body language and vocal communications (notably growling) that help them retain resources from others or abandon them without injury (81). With practice, some dogs may learn to guard chews, which can later enable them to displace others from chews and related resources. Dogs innately assess their resource-holding potential (RHP); a dog lacking the resource requires a higher potential to challenge the holder due to the gaming advantage (187). The protagonist’s growl is acoustically specific for keeping a rival dog at bay (188). These behaviours help prevent outright fights; typically, participants with lower RHP will withdraw. Only dogs with similar RHP may escalate the interaction (187). This may, at least in part, explain why inter-dog aggression is most common between dogs where the aggressor is, on average, only 1.5 kg heavier than the defender (189) and between iso-sexual (especially female) dyads (81). Agonistic interactions contribute to the complexity of social living, often mitigated by the benefits of companionship and play.

Chewing may be a socially facilitated behaviour in that its performance increases the probability of observing conspecifics performing the same behaviour (190). In the same way, puppies will eat more in a set period when eating in groups compared to solitary eating (191, 192), and adult dogs perform predatory sheep-chasing more when with another chasing dog than when alone (193), both indicators of social facilitation and appetitive behaviours. Anecdotally, socially facilitated chewing appears to occur with both conspecifics and human guardians, as dogs appear to enjoy “joining in” when humans eat or when other dogs are chewing.

Chewing facilitates the expression of hunting behaviours through prehension, biting, and ripping, providing engagement and stimulation with prey, such as other animals or replicas. Companion dogs may never have the opportunity to display these aspects of hunting and food acquisition behaviour within a dog’s telos. The absence or denial of hunting tropes can be frustrating, particularly for certain behavioural phenotypes.

#### 4.4.3 Interactions with humans

Human carers observe the enriching and health benefits of chewing options in the lives of their companion or kennelled dogs. They can serve as a chew source and may be offered as treats. Arhant (139) found that many owners report chewing keeps dogs occupied, calms them, and distracts them from unpleasant experiences. These observations help explain the popularity of chews among dog owners. Howell et al. (194) found that 67% of respondents provided dental chews or toys, while 64% gave bones.

The predictability of daily provision, particularly on a fixed interval schedule, can foster calm and positive behaviour. Predictability is a crucial aspect of managing captive animal environments; for example, the scheduled arrival of a chewing opportunity can lead to positive anticipatory behaviours that (195) indicate a positive affective state (161, 196) and aid in relaxation. However, an abundance of choice with multiple chewable items available is typical in normal dog dens.

Chews can increase the risk of conflict with humans when they misunderstand the value that a dog may ascribe to the chew and overlook the strong motivation to protect it. Human ignorance of the benefits of having such items available can result in valuable items being “cleaned up” or removed prematurely. As discussed in the context of conspecifics, a dog that has experienced the loss of valued resources and is not ingested quickly (as noted above with conspecifics) may chew less and swallow prematurely, leading to GIT injury or illness.

Humans control how often dogs receive chews. Chews provided by a caregiver have the potential to strengthen the human–dog bond, as dogs may form positive associations with those who provide such a valuable item. When considered a natural part of daily life that meets the canine telos to chew, limiting access to chews limits a dog’s ability to express this element of the canine ethogram. Dogs often like to chew on aged and partially degraded bones or chews. When humans remove these items in a bid to “tidy up,” their actions can have unintended consequences. For example, denying free access to chewing opportunities may diminish potential positive states, such as the freedom to return to the item and chew it at leisure.

Furthermore, doing so may reduce the comfort that dogs experience in a preferred canine environment, which naturally includes familiar chews, both cached and overt. Access to chewable items can increase the risk of agonistic interactions with others. Therefore, some logistical hurdles may need to be addressed to provide dogs with the benefits of chewing without disrupting social bonds. The role of human expectations and education may be significant.

### 4.5 Mental state

The fifth domain is the animal’s mental state, which is affected by the four physical domains. We propose that chewing is crucial for dogs. This is demonstrated by the anticipatory behaviour exhibited (184) when chewing is imminent, the focus and positive body language directed toward the item when it comes into view, and perhaps above all, the defence of the resource once acquired. A chew that is actively protected by growls and bites momentarily holds more value for the dog than the bond it shares with its conspecific group members or its human carer. For a species as social as *Canis familiaris*, this motivation highlights the extraordinary value of chewing and conversely suggests that the lack of opportunity compromises welfare through deprivation (197). Furthermore, the ability to chew when the impulse arises is a prominent indicator of positive welfare, suggesting (198) that chews in the environment may provide positive welfare even when not in active use.

Freedom from pain, harm, and other negative affective states is one aspect of welfare. However, beyond sparing animals from suffering, human caregivers of sentient beings have a further obligation: to ensure that dependent animals experience happiness and flourish (6, 199). Telos encompasses the “dogness” of being a dog and includes all the behaviours exhibited by free-roaming dogs over phylogeny (199), such as sniffing, chewing, and digging. It reveals the fulfilment, satisfaction, and happiness that can be attained from the opportunity to gnaw, savour, and manipulate items. Given the net accumulation of affects discussed in this review, the benefits of chewing for the mental state are both immediate (during the act of chewing) and enduring when an environment is enriched with chews and chew choices. Conversely, since chewing is a telos of dogs and contributes to positive welfare when provided, a degree of negative affect is expected from deprivation or minimal provision of chew items. Whether a dog that has never had a chew misses the opportunity to chew is a moot point. However, when considering telos, the denial of most innate rewarding behaviours will leave the motivation to chew unmet, thus increasing frustration.

The topic of chewing can evoke many responses from dog carers, the majority of which are positive and some negative. The latter is more likely when chews trigger agonistic interactions with conspecifics and humans or lead to adverse health effects. Understanding the dog’s social structure and body language, as well as managing the environment with the individual dog in mind, can help alleviate any negative outcomes associated with chewing. However, this highlights the anthropocentric distortion that can prevail when considering what constitutes good welfare, even for beloved companion dogs, which are, in essence, subject to the decisions of their human carers (200). Overall, using the Five Domains lens to examine chewing in dogs provides insight into the impact that the opportunity to chew has on a dog’s psychological welfare.

## 5 Discussion

The goal for this article is to integrate current knowledge on chewing in dogs to stimulate thought and develop research questions through a biological lens (Tinbergen’s questions) and welfare science (the Five Domains framework). Given that chewing promotes positive welfare states in dogs and is a part of telos, carers are responsible for allowing dogs the opportunity to engage in this behaviour whenever it is safe. Negative effects resulting from chewing are rare but are typically associated with undesirable interactions with people and other animals or with gastrointestinal injury, which must be weighed against the consequences of deprivation. These risks highlight that the provision of chews is most effective when tailored to individual needs and warrants further research into the chewing abilities and effects of various types of chews based on morphology. This research will help determine whether the increased prevalence of dental disease in smaller and older dogs is related to the kinds of chews these dogs are provided with and how chews can be offered to enhance dental health while ensuring safety. Further exploration into whether chewing foods influence oral microbiome health more significantly than indigestible chews would enrich our understanding of dental hygiene.

There is merit in researching the time spent chewing by companion and kennel dogs when given the opportunity, as well as what constitutes minimal provision of chews. This review highlights the importance of chewing for a dog’s welfare and suggests future work to determine whether there is a difference between the motivation to chew for nutrition and the motivation to chew for enrichment, which would be valuable. Research should be conducted to identify the optimal match of chewable substrates to reduce negative sequelae, inform veterinarians and canine professionals, and provide recommendations to human carers, allowing dogs to enjoy the many potential benefits of ad libitum chewing.

The current review process has limitations in that it is biassed toward empirical data, as we considered only peer-reviewed articles and minimal grey literature or protocols from industry sources. While some articles in the dataset reported outcomes from practical contexts, most findings derive from research settings. Therefore, information regarding dog owners’ observations of their dogs’ chewing in real-world contexts may be underrepresented, as these observations are infrequently reported in the scientific literature. Future research that includes behavioural observations from domestic settings would bridge the gap between scientific and practical knowledge.

The review provides a snapshot of relevant anglophonic literature but does not encompass all of the available literature. Data were gathered from PubMed, Google Scholar, and the University of Sydney library database. The authors acknowledge that these search parameters may restrict or prohibit access to some research outputs. Many subject areas mentioned were not necessarily the primary focus of the published report. Information in these fields was provided as supplementary information and may be simplified.

## 6 Conclusion

In summary, for dogs, chewing plays a crucial role across all four of Tinberg’s questions: functionally, evolutionarily, mechanistically, and ontogenically. The literature indicates that chewing promotes dental and oral hygiene, is an integral component of digestion, and is important for GIT function due to its association with saliva production, which promotes gut motility and maintains mucosal integrity. Chewing has a positive impact on the oral microbiome and influences the colonic microbiome. Outside the GIT, chewing helps moderate and regulate arousal by alleviating stress and downregulating responses, even during pregnancy, providing lifelong behavioural benefits to unborn puppies. In dogs, as in humans and mice, chewing likely promotes memory consolidation and is linked to bone and cognitive health. Through these biological processes, chewing positively affects individuals throughout all life stages, from conception to senescence.

The value of chewing to positive dog welfare reflects its importance in all four domains of welfare and, overall, positively affects the fifth. Using the Five Domains Framework has demonstrated the welfare benefits of chewing for dogs and confirmed that they largely outweigh any negative consequences.

Chewing can alleviate negative states, such as frustration, depression, and boredom, and mitigate the effects of separation. Similarly, as part of their natural behaviour, it may induce positive states such as engagement and focus, as well as feelings of energy and calmness, and promote the pleasure of autonomy and the expression of normal chewing behaviour. Overall, dogs are primarily motivated to chew because it supports their wellbeing and fitness. Dogs have the physical ability to chew and gain numerous health and welfare benefits, which depend on how chews are provided, as well as individual temperament, substrate type, and environment. Furthermore, opportunities for chewing should not be restricted, as dogs have evolved with largely unrestricted access to chewable items and the freedom to chew at will. The quantity and quality of chewing required for optimal health and wellbeing outcomes in dogs are not well understood, and we must balance the potential risks of under-provision or deprivation against the considerable benefits to dogs’ health and welfare.

## Footnotes

^1^Based on GREENIES™ Original Large Dental Dog Treat, P 27.6%, F 14.5%, CHO 57.9% by omission and calculated based on macronutrient ratio by energy (https://www.greenies.com.au/products/dog-products/greenies-original-large-dental-dog-treats-170g-4-treats-large)

^2^Based on PEDIGREE® DentaStix™ Daily Oral Care Large Dog Treat, P 6.8%, F 3.7% CHO 89.4% by omission based on macronutrient ratio by energy (https://www.pedigree.com.au/our-products/dental-treats/pedigree-dentastix-daily-oral-care-large-dog-treat-triple-action-dental)

^3^Dr. B’s BARF diet, Real Pet Food Company, Crows Nest, NSW, Australia.

^4^Pet Food Industry Association of Australia (PFIAA) and Association of American Feed Control Officials (AAFCO).
